# Targeting intracellular proteins with cell type-specific functions for cancer immunotherapy

**DOI:** 10.1093/lifemedi/lnad019

**Published:** 2023-06-17

**Authors:** Madison E Carelock, Rohan P Master, Myung-Chul Kim, Zeng Jin, Lei Wang, Chandra K Maharjan, Nan Hua, Umasankar De, Ryan Kolb, Yufeng Xiao, Daiqing Liao, Guangrong Zheng, Weizhou Zhang

**Affiliations:** Department of Pathology, Immunology and Laboratory Medicine, College of Medicine, University of Florida, Gainesville, FL 32610, USA; Cancer Biology Concentration, Biomedical Graduate Program, College of Medicine, University of Florida, Gainesville, FL 32610, USA; Department of Pathology, Immunology and Laboratory Medicine, College of Medicine, University of Florida, Gainesville, FL 32610, USA; Department of Pathology, Immunology and Laboratory Medicine, College of Medicine, University of Florida, Gainesville, FL 32610, USA; UF Health Cancer Center, University of Florida, Gainesville, FL 32610, USA; Diagnostic Laboratory Medicine, College of Veterinary Medicine, Jeju National University, Jeju 63243, Republic of Korea; Research Institute of Veterinary Medicine, College of Veterinary Medicine, Jeju National University, Jeju 63243, Republic of Korea; Department of Pathology, Immunology and Laboratory Medicine, College of Medicine, University of Florida, Gainesville, FL 32610, USA; Cancer Biology Concentration, Biomedical Graduate Program, College of Medicine, University of Florida, Gainesville, FL 32610, USA; Department of Pathology, Immunology and Laboratory Medicine, College of Medicine, University of Florida, Gainesville, FL 32610, USA; Immunology Concentration, Biomedical Graduate Program, College of Medicine, University of Florida, Gainesville, FL 32610, USA; Department of Pathology, Immunology and Laboratory Medicine, College of Medicine, University of Florida, Gainesville, FL 32610, USA; Department of Pathology, Immunology and Laboratory Medicine, College of Medicine, University of Florida, Gainesville, FL 32610, USA; Department of Pharmacodynamics, College of Pharmacy, University of Florida, Gainesville, FL 32610, USA; Department of Pathology, Immunology and Laboratory Medicine, College of Medicine, University of Florida, Gainesville, FL 32610, USA; Department of Pathology, Immunology and Laboratory Medicine, College of Medicine, University of Florida, Gainesville, FL 32610, USA; UF Health Cancer Center, University of Florida, Gainesville, FL 32610, USA; Department of Medicinal Chemistry, College of Pharmacy, University of Florida, Gainesville, FL 32610, USA; UF Health Cancer Center, University of Florida, Gainesville, FL 32610, USA; Department of Anatomy and Cell Biology, College of Medicine, University of Florida, Gainesville, FL 32610, USA; UF Health Cancer Center, University of Florida, Gainesville, FL 32610, USA; Department of Medicinal Chemistry, College of Pharmacy, University of Florida, Gainesville, FL 32610, USA; Department of Pathology, Immunology and Laboratory Medicine, College of Medicine, University of Florida, Gainesville, FL 32610, USA; Cancer Biology Concentration, Biomedical Graduate Program, College of Medicine, University of Florida, Gainesville, FL 32610, USA; UF Health Cancer Center, University of Florida, Gainesville, FL 32610, USA; Immunology Concentration, Biomedical Graduate Program, College of Medicine, University of Florida, Gainesville, FL 32610, USA

**Keywords:** multifunctional protein targets, immune checkpoint inhibitors, immunotherapy, tumor microenvironment (TME)

## Abstract

Immune checkpoint inhibitors (ICIs) use antibodies that block cell surface immune checkpoint proteins with great efficacy in treating immunogenic or “immune hot” tumors such as melanoma, kidney, and lung adenocarcinoma. ICIs have limited response rates to other non-immunogenic cancers. The tumor microenvironment (TME) consists of many cell types that collectively promote tumor progression. Cancer therapeutics are commonly designed to target one molecule in one defined cell type. There is growing evidence that long-term therapeutic responses require the targeting of cancer cells and tumor-promoting populations within the TME. The question remains whether we can identify targetable molecules/pathways that are critical for multiple cell types. Here, we will discuss several molecular targets that may fit a “two or multiple birds, one stone” model, including the B-cell lymphoma-2 (BCL-2) family pro-survival factors, transcriptional factors including signal transducer and activator of transcription 3, the nuclear receptor 4A family (NR4A1, NR4A2, and NR4A3), as well as epigenetic regulators such as bromodomain and extra-terminal (BET) family proteins, histone deacetylase family, SET domain bifurcated histone lysine methyltransferase 1 (SETDB1), and lysine-specific demethylase 1 (LSD1/KDM1A). We will focus on the rationale of these targets in immune modulation, as well as the strategies for targeting these important proteins for cancer therapy.

## Introduction

Current cancer immunotherapy focuses on the use of immune checkpoint inhibitors (ICIs)—antibodies that block the function of immune checkpoints namely cytotoxic T-lymphocyte-associated protein 4 (CTLA-4), programmed cell death protein 1 (PD-1), programmed cell death 1 ligand 1 (PD-L1), or lymphocyte activation gene 3 (LAG3) [1–3]. Many other antibodies that block newly discovered immune checkpoints are in the development pipeline as well [4]. While ICIs have significantly improved the outcomes in patients with immunogenic cancer types, the majority of cancer patients with non-immunogenic cancers—such as most breast cancers or squamous lung carcinomas—exhibit a very limited response rate to ICIs [1]. In addition to those cases where *de novo* resistance to ICIs occurs due to the nature of cancer pathology, acquired resistance, and super progressors are also common in cancers that initially respond to ICIs. The resistance mechanisms have been explored and discussed in several outstanding review articles [1, 5]. The purpose of this review is to explore the potential of targeting intracellular proteins that have distinct functions within different cell types, which provides rationales to potentially benefit cancer patients with ICI-resistant cases.

**Figure 1.**
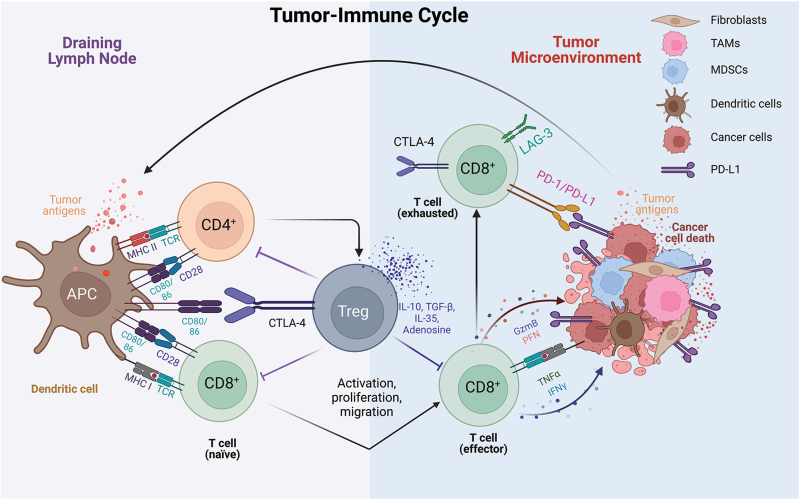
**The tumor-immune cycle.**The tumor-immune cycle consists of two-way trafficking between the TME and the draining lymph nodes (DLN). Tumor cells release tumor antigens via inflammatory cell death, or other forms of cell death, which can be endocytosed by antigen-presenting cells (APCs). In the DLN following antigen processing, APCs present antigenic peptides on the major histocompatibility complex (MHC) to activate naïve CD4^+^ and CD8^+^ T cells by interacting with the T cell receptors (TCRs) (Signal 1). Additionally, T cell activation requires co-receptor stimulation between CD80/CD86 and CD28 to reprogram naïve phenotypes to effector T cell phenotypes. Effector T cells then go through clonal expansion and migrate to the TME where they secrete effector molecules such as granzyme B (GzmB), perforin (PFN), tumor necrosis factor α (TNFα), and interferon γ (IFNγ). Along this process, effector T cells may become exhausted as characterized by the overexpression of cell surface markers such as PD-1, CTLA-4, and/or LAG-3. Immunosuppressive cell populations within the TME, such as the tumor cells, myeloid-derived suppressor cells (MDSCs), and tumor-associated macrophages (TAMs), express PD-L1 to interact with PD-1 to inhibit the function of CD8^+^ effector T cells. Furthermore, regulatory T cells (Tregs) also express CTLA-4 and secrete IL-10, IL-35, transforming growth factor β (TGF-β), and adenosine metabolites to suppress effector T cell or APC function and promote tumor progression.

The tumor microenvironment (TME) consists of many cell types. Within advanced tumors, these cell types—if not interfered with—cooperate to create an immune suppressive TME and promote tumor progression [6]. This article will mainly focus on the immune modulatory roles of these proteins that have varying yet crucial functions within different cancer and immune cell types. We will not thoroughly discuss fibroblasts or endothelial cells within the TME since their roles in cancer development and metastasis have been well-established in previous literature [7–13]. To initiate the classic tumor-immune cycle (**Fig. 1**) [14, 15], tumor cells need to release tumor antigens—including some self-antigens, neoantigens, or endogenous retroviral (ERV) antigens—to antigen-presenting cells (APCs). Dendritic cells (DCs) are professional APCs and can cross-present tumor antigens to naïve CD4^+^ T cells and CD8^+^ T cells within secondary lymphoid organs, mainly in the draining lymph nodes. After priming, activated CD4^+^ T cells can produce inflammatory cytokines that further stimulate CD8^+^ T cells to promote their cytolytic effector function and migration to the TME [15]. Antigen presentation may also occur in tertiary lymphoid structures within the TME [16]. T cell priming and activation within lymphoid tissues depend on several signals. Signal 1: the interaction between antigen being presented by the major histocompatibility complex (MHC) and the T cell receptors (TCR); Signal 2: the co-stimulation (CD80/86) to CD28; and/or Signal 3: stimulation by other co-stimulatory signals and cytokines, which can be provided from other immune cells such as CD4^+^ T cells [14, 15]. Ipilimumab is one ICI that targets and inhibits CTLA-4, a molecular that has a much higher binding affinity to CD80/CD86 than CD28. Relatlimab is another ICI that targets LAG-3, an immune checkpoint known to inhibit MHC/TCR signaling [1, 2]. Activated T cells can then migrate from lymph nodes to the TME where they can kill target cancer cells in an antigen-dependent manner, though bystander killing can occur. However, the TME is generally immune suppressive by many different mechanisms including immature vasculature, the enrichment of immune suppressive cell types such the regulatory T cells (Tregs) and myeloid-derived suppressor cells (MDSCs), the overexpression of immune suppressive cytokines such as transforming growth factor β (TGFβ), IL-35, IL-10, immune exclusive extracellular matrix, and the expression of immune checkpoints (e.g., PD-1/PD-L1) [14, 15].

Most immune checkpoints, including CTLA-4, PD-1, and PD-L1 with antibodies approved by the Food and Drug Administration (FDA), are expressed in exhausted T cells (T_exh_) that have encountered tumor antigens [14, 15]. ICIs work to reinvigorate these tumor antigen-specific T cells [14, 15]. In some cases, clonal replacement has been proposed to be the major mechanism of action for ICIs, where ICIs are proposed to eliminate exhausted tumor-infiltrating (TI) T cell clones, allowing for the recruitment and clonal expansion of novel tumor-specific T cell clones with improved tumor-killing activity [17]. Nonetheless, current ICIs are believed to work at the very end stage of the T cell activation process during the tumor-immune cycle when T cells have already been activated and/or become exhausted. The earlier stages of the tumor-immune cycle [14, 15]—including tumor antigen production and presentation by APC, T cell priming and expansion, or lysis of tumor cells by T cells—are emerging points of action during the process of clinical translation. Another major limitation of antibody-based ICIs is the surface localization of target proteins, which excludes all cytoplasmic or nuclear proteins. Many intracellular proteins have immune inhibitory functions similar to those of immune checkpoints and can broaden therapeutic opportunities since these proteins can be present at each step of the tumor-immune cycle.

In addition to the T_exh_ as therapeutic targets of ICIs, many other cell types can potentially be targeted within the TME. These cells include tumor-associated macrophages (TAMs), MDSCs, and Tregs—all of which can directly promote tumor progression or indirectly maintain an immune-suppressive TME. The following sections will briefly discuss some of these targetable cell types central to this review, with an emphasis on emerging molecular targets that may have a distinct function within different immune cell types.

TAMs are an integral component of the TME and can be very abundant in tumors. There are many outstanding reviews related to TAMs [18–21]. TAMs play many essential roles during tumor progression including, but not limited to, directly inducing cancer cell proliferation/invasion, metastasis, angiogenesis, extracellular matrix remodeling, and immune modulation [18–21]. TAMs are generally considered to contribute to an immune suppressive TME via aberrant regulation of proinflammatory/inhibitory cytokines (e.g., IL-10, TGF-β, arginase, indolamine 2,3-dioxygenase (IDO), prostaglandins, and IL-1β), the expression of immune checkpoints (e.g., PD-L1, PD-L2, and B7-H4), or the direct or indirect influence on other immune suppressive cell types. For example, TAMs can induce Tregs, MDSCs, and/or T cell exhaustion [19]. In addition to facilitating the natural progression process, TAMs have been linked to therapeutic responses to different treatment regimens including chemotherapy and ICI-based immunotherapy [19].

MDSCs are pathologically activated granulocytes (gMDSCs) or monocytes (mMDSCs) with potent immune suppressive capacity [22–24]. The accumulation of IDO or chemokines such as C-C motif chemokine ligand 2 (CCL2), and C-C motif chemokine ligand 5 (CCL5) [25, 26] contributes to the recruitment of MDSCs to the TME. Cytokines within the TME such as granulocyte colony stimulatory factor (G-CSF), stem cell factor (SCF), granulocyte-macrophage colony-stimulating factor (GM-CSF), and macrophage colony-stimulating factor (M-CSF) stimulate myelopoiesis and proliferation of MDSCs [22, 23]. MDSCs drive tumor angiogenesis, growth, progression, and metastasis through both immune-dependent and immune-independent mechanisms [23, 26]. MDSCs contribute to the suppressive nature of the TME through the release of molecules, such as arginase-1 (ARG1) and reactive oxygen species (ROS) [22, 27], which inhibit T cell activation and effector function. MDSCs sequester crucial metabolic molecules, such as cysteine [27], required for T cell activation and effector function. MDSCs can secrete vascular endothelial growth factor to promote neovascularization [25] within the TME to support tumor growth and progression and recruit additional MDSCs to the TME [27]. MDSCs contribute to the early stages of metastasis through the secretion of matrix metalloproteinase 9 to induce vascular permeability and diminish the extracellular matrix of cancer cells, allowing their dissemination from the primary tumor [28]. The immune suppressive and extracellular remodeling functions of MDSCs make them important facilitators of metastasis. The development of immunotherapy to target the pro-tumor mechanisms of MDSCs may be a viable solution not only to reduce the suppression of T cells but also to diminish the growth and metastasis of the tumor.

Tregs are one of the major immune suppressive cell types and are central to the maintenance of immune homeostasis under physiological conditions. Tregs require activation of their TCR to become suppressive; however, after activation, their suppressive effector function is not necessarily specific to the effector T cells recognizing the same antigen [29]. Tregs demonstrate tumor and neoantigen reactivity and can be activated and expanded within the TME [30]. Studies have also highlighted the presence of Tregs in the peripheral blood that are specific to tumor antigens and may facilitate metastasis [30]. Tregs can elicit their suppressive effector function through a variety of mechanisms including the production of immunosuppressive cytokines (e.g., IL-10, TGF-β, and IL-35), increasing expression of suppressive surface-protein molecules (e.g., CTLA4 and PD-1), and sequestering IL-2 through CD25, a high-affinity IL-2 receptor [31, 32]. Tregs secrete numerous potent inhibitory cytokines that inhibit the anti-tumor effector function of immune cells within the TME [33] and can directly induce tumor progression or metastasis [34]. The increased expression of inhibitory immune checkpoint molecules acts to diminish the anti-tumor immune response within the TME by interfering with the activation of T cells. For example, CTLA-4 expressed on tumor-infiltrating Treg cells (TI-Tregs) can bind to CD80/86 with higher affinity than CD28, impairing the maturation of APCs and costimulatory signals to further hinder the activation of anti-tumor CD4^+^ and CD8^+^ T cells. PD-1, LAG-3, and TIM-3 also negatively regulate the function of CD4^+^ and CD8^+^ T cells, ultimately promoting Treg proliferation and immune suppressive function [35]. Tregs can also participate in crosstalk with cancer cells, stromal cells, and other inhibitory immune cells in the TME to further promote anti-tumor immune evasion and therapeutic resistance [35]. Due to these activities, Tregs remain a major obstacle in anti-tumor immunity and therapy. Developing an effective therapy to target and modulate Tregs can switch the TME from immune suppression to the induction of the host’s anti-tumor response.

Based on the above considerations, here we will discuss several families of proteins involved in both cancer progression and immune modulation based on the following criteria: (i) they should have critical functions in cancer cells and immune suppressive cell populations such as Tregs, T_exh_ MDSCs, etc.; (ii) therapeutic targeting of these molecules will not significantly impair anti-tumor effector function of major immune cells such as effector T cells or natural killer cells; and/or (iii) they should be targetable with known inhibitors/degraders with limited off-target effects and/or toxicity. While there are many proteins that fit the above criteria, we chose several representative proteins in each category for further discussion, including (i) cytoplasmic proteins: the B-cell lymphoma (BCL)-2 (BCL-2) family pro-survival factors (BCL-2 and BCL extra-large (BCL-X_L_)), (ii) transcription factors: STAT3 and nuclear receptor 4A (NR4A) family, and (iii) epigenetic modulators: bromodomain and extra-terminal (BET) family proteins, HDAC family, SETDB1, and LSD1/KDM1A. We chose these proteins based on research that has extensively validated these targets for cancer therapy and from which multiple inhibitors/degraders have been studied and optimized both structurally and functionally. We purposely choose these targets due to their emerging roles in the immune modulation within the TME, the representation of different categories, and the availability of local expertise. Depending on the nature of these “intracellular immune checkpoints,” small molecule inhibitors can be used for targets with enzymatic activities; or targeted protein degraders—including molecular glues and proteolysis-targeting chimeras (PROTACs)—can be used for both enzymatic targets and traditionally believed non-targetable proteins. Most importantly, some of these proteins already have inhibitors/PROTACs in advanced clinical trials or with FDA approval that can be repurposed for immune modulation during cancer therapy.

## The BCL-2 anti-apoptotic proteins

**Figure 2.**
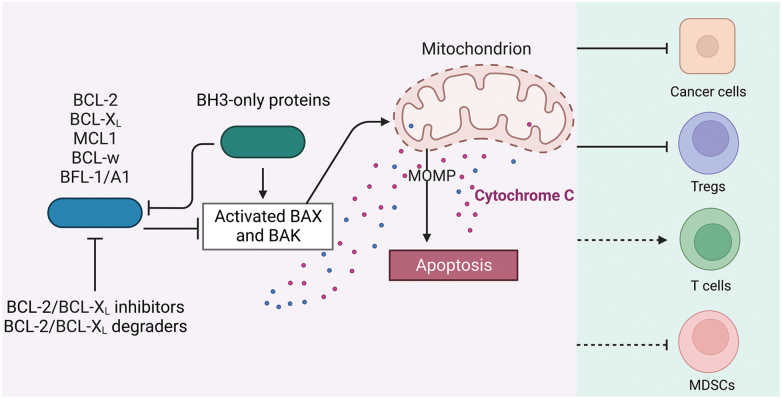
**The anti-apoptotic BCL-2 family members in immune regulation and cancer therapy.**The anti-apoptotic BCL-2 family includes BCL-2, BCL-X_L_, MCL1, BCL-w, and BFL-1/A1. These proteins can prevent apoptosis of cells by counteracting BH3-only proteins or BAX/BAK proteins. BCL-2 and BCL-X_L_ are the most actively targeted members for cancer therapy with a series of inhibitors/degraders available, which can not only promote the apoptosis of cancer cells but also some immune suppressive cells such as tumor-infiltrating (TI)-Tregs or MDSCs. Green shade indicates the influence of BCL-2/X_L_ inhibitors/degraders on different immune cell types via direct activation/inhibition (solid line) or indirect activation/inhibition (dashed line) [44–46].

Anti-apoptotic BCL-2 family members, including BCL-2 and BCL-X_L_, are well-known for their anti-apoptotic functions [36, 37]. BCL-2 family members play a major role in regulating types of cell death, including apoptosis, necrosis, and autophagy. There are three primary subfamilies which include anti-apoptotic proteins (BCL-2, BCL-X_L_, BCL-w, MCL-1, and BFL-1/A1), pro-apoptotic proteins (BAX, BAK, and BOK), and pro-apoptotic BCL-2 homology 3 (BH3)-only proteins (BAD, BID, BIK, BIM, NOX, PUMA, etc.). The pro-apoptotic BH3-only proteins can be further divided into activator and sensitizer proteins. The activator BH3-only proteins can bind to the BH3 domain on BAX and BAK to activate them which results in BAX/BAK oligomerization and forms the pores in the mitochondrial outer membrane that ultimately regulates mitochondrial outer membrane permeabilization (MOMP). This allows the release of apoptogenic factors, such as cytochrome c, from the mitochondrial intermembrane space that leads to the activation of the caspase cascade. Once activated, caspases can cleave several structural proteins vital for normal cellular functions, and result in the formation of apoptotic blebs that can be phagocytosed by macrophages and dendritic cells. Anti-apoptotic proteins typically regulate this process by binding to the BH3 domain of BAX/BAK which inhibits their function via sequestration. The sensitizer BH3-only proteins can bind to the BH3 domain of anti-apoptotic proteins to inactivate them, which promotes apoptosis [36, 38]. Evading apoptosis is one of the earliest requirements for cancer progression and one of the best-established hallmarks of cancer [39, 40]. Cancer cells traditionally upregulate BCL-2 anti-apoptotic proteins which provide them with resilience against stimuli, such as growth factor deprivation, hypoxia, and oxidative stress, that typically induce apoptosis in cells. Many cytotoxic cancer drugs depend on BAX/BAK-dependent mechanisms to facilitate their anti-tumor effects, and upregulation of BCL-2 anti-apoptotic proteins facilitates resistance to several chemotherapies (Fig. 2) [36, 37, 39–41]. The dependence of cancer cells on BCL-2 family proteins makes them outstanding targets for targeted cancer therapy. The anti-apoptotic BCL-2 proteins can be counterbalanced by proapoptotic BH3-only BCL-2 proteins; as such, the BH-3 mimetics were developed to promote apoptosis in cancer cells (**Fig. 2**) [36, 42, 43].

ABT-737 is the first BH3 mimetic [43, 47] and ABT-263 (navitoclax) is an orally bioavailable alternative [48]; both compounds inhibit the anti-apoptotic functions of BCL-2, BCL-X_L,_ and BCL-w [43, 47, 48]. ABT-263 entered a phase I clinical trial as a single agent and had some level of clinical responses [49]. Several other phase I trials confirmed the efficacy of ABT-263, but patients exhibited signs of thrombocytopenia and T-cell lymphopenia due to the inhibition of BCL-X_L_ and BCL-2, respectively [50–52].

To reduce the on-target toxicity of the earlier generation of inhibitors, ABT-199 (venetoclax), a BCL-2-specific inhibitor, was developed and can potently induce apoptosis in BCL-2-dependent cancer cells, but not BCL-X_L_-dependent cancer cells [53]. Venetoclax has been approved by the FDA and other countries for the treatment of several blood cancers [54, 55]. Venetoclax exhibits low toxicity to platelets, which is consistent with no sign of thrombocytopenia in patients; the dose-limiting toxicity to neutrophils and certain cases of tumor lysis syndrome still occur but can be largely managed by changing the administration regimen [56]. In addition to the direct killing of cancer cells, a recent article demonstrated that venetoclax increases the number of tumor-infiltrating effector memory T cells, which further sensitizes tumors to ICI therapy [57]. The direct effect on T cells by venetoclax is likely due to the compensation from BCL-X_L_ upregulation [57] as well as the direct enhancement of T cell effector function by venetoclax via inducing ROS through the inhibition of respiratory chain complex formation [58]. Chimeric antigen receptor T-cell (CAR-T) immunotherapy has been very successful in B cell lymphomas and leukemias, but more than 60% of these patients don’t respond or relapse. In a targeted screening including a library of 29 proapoptotic drugs, venetoclax was able to enhance the tumor-killing effect from CAR-T cells targeting CD19^+^ B cell lymphomas, further supporting the direct activation of T cells by venetoclax [59].

**Figure 3.**
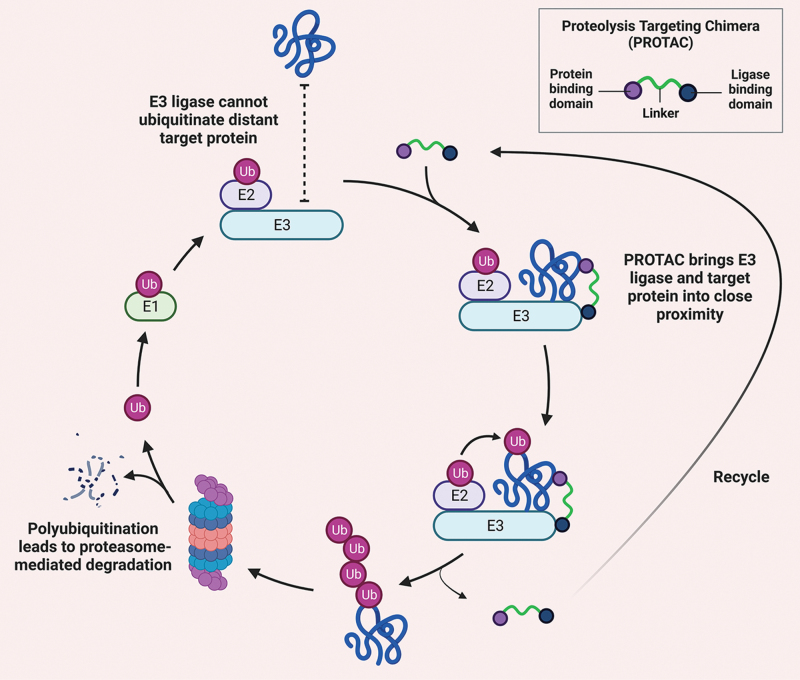
**Schematic of PROTAC mechanism of action.** PROTACs function through the recruitment of a specific E3 ligase to the protein of interest (POI). This is accomplished by linking a ligand for the POI and another ligand for an E3 ligase such as CRBN or VHL. The two ligands are joined by a chemical linker moiety. With full functionality, the PROTAC molecule brings the POI and E3 ligase into proximity, which allows the ubiquitin ligase complex to attach a polyubiquitin chain to the POI, labeling it for degradation via the proteasome.

BCL-X_L_-specific inhibitors—including WEHI-539 [60], A-1155463^60^, and A-1331852^61^—have also been developed to target cancer types that depend on BCL-X_L_ for survival with reduced toxicity relative to ABT-263. The common dose-limiting toxicity to platelets remains, which limits these inhibitors to a very narrow therapeutic window with none being approved for cancer therapy [60, 62]. Zheng and Zhou groups recently developed several BCL-X_L_-specific degraders using PROTACs [44, 63, 64], a 20-year-old technology that has revolutionized targeted-protein degradation since its conception in 2001 [65]. These heterobifunctional molecules comprise two ligands connected by a linker. PROTACs function by recruiting their target proteins and an E3 ubiquitin ligase so that the target protein is polyubiquitinated and subject to proteasome-mediated degradation (**Fig. 3**). PROTAC-based degraders offer multiple advantages over traditional small-molecule inhibition systems, especially regarding drug dosage, resistance, side effects, and targeting of “undruggable” molecules [66]. In addition, E3 ligases may exhibit tissue specificity for potential targeting of cancer cells while sparing other healthy cells. For example, the BCL-X_L_-specific PROTAC DT2216 from the Zhou and Zheng groups can lead to BCL-X_L_ degradation in cancer cells but not in platelets due to the low expression of the von Hippel-Lindau (VHL) E3 ligase in platelets [44, 63, 64]. The superior selectivity of DT2216 in degrading BCL-X_L_ in cancer cells makes it a less toxic drug to treat BCL-X_L_-dependent cancers. DT2216 is currently in phase I clinical trial (ClinicalTrials.gov Identifier: NCT04886622), which is an open-label, first-in-human, dose escalation study in subjects with histologically or cytologically confirmed advanced or metastatic malignancies who are no longer responsive to approved or accepted standard-of-care interventions. Our follow-up study showed that tumor-infiltrating (TI)-Treg cells have elevated expression of BCL-X_L_, at much higher levels than Treg cells from paired blood or effector T cells from tumors or blood [45]. More importantly, DT2216 or PZ15227—a cereblon (CRBN) E3 ligase-based BCL-X_L_ PROTAC—leads to efficient degradation of BCL-X_L_ in TI-Tregs and the reduction of TI-Tregs. We also found that TI-DCs may also depend on BCL-X_L_ for survival as DT2216 reduces the number of TI-DCs, but the reduction in TI-DC numbers did not seem to negatively influence T cell activation since we observed a significant increase in granzyme B^+^ active CD8^+^ T cells [45]. Another report showed that BCL-X_L_ is elevated in TI-MDSCs and inhibition of BCL-X_L_ by ABT-737 leads to the spontaneous apoptosis of MDSCs in tumor-bearing mice [46]. All these results strongly support that BCL-X_L_ PROTACs are promising cancer therapeutics that kill cancer cells directly, as well as shift the TME to a more immune-active state (**Fig. 2**). Though the direct cancer cell-killing is not sufficient to reduce tumor growth in several cancer models [44, 63, 64], it may provide more tumor antigens for cross-presentation to initiate the tumor-immune cycle [14, 15], and/or sensitize cancer cells to other cytotoxic agents or active CD8^+^ T cell-mediated killing. Thorough studies are warranted to explore the potential of BCL-X_L_ PROTACs in immune modulation and in combinatory therapy with chemotherapies, radiotherapies, or immunotherapies.

### Conclusive remarks:

BCL-2 family proteins have proven to be outstanding targets for cancer therapy, but their importance in controlling normal cell survival often leads to dose-limiting on-target toxicity of BCL-2 inhibitors that induce neutropenia or diarrhea, or BCL-X_L_ inhibitors that result in thrombocytopenia. DT2216—the first BCL-X_L_ PROTAC in the phase I trial—exhibits reduced toxicity to platelets due to the low expression of VHL E3 ligase in platelets. It is still unknown whether BCL-2-targeting PROTAC can sufficiently reduce the toxicity to neutrophils or gastric intestinal cells. As the choice of E3 ligases can significantly reduce dose-limiting on-target toxicity of DT2216, it provides the rationale to choose tissue-specific or cancer-type-specific E3 ligases for PROTAC synthesis. The field will greatly benefit from accumulating more small molecule ligands for the known E3 ligase pool for tissue selectivity. A complete understanding of the mechanisms of action for currently available drugs will further guide the rational design of combinatory therapies. Cancer cells, in particular solid cancers, develop various mechanisms to evade apoptosis. For example, most solid cancer cells cannot be efficiently killed by BCL-X_L_ or BCL-2 inhibition, but their inhibition may sensitize cancer cells to various chemotherapy or immunotherapy. These synergistic effects can be harvested to treat cancers in a more efficient way. Immune modulations by pharmacological BCL-X_L_ or BCL-2 inhibition can be very different from what we know based on genetic or biological data from the literature. It has been known for decades that CD8^+^ T cells rely on BCL-2 for survival, but venetoclax treatment has been shown to promote the activity of TI-CD8^+^ T cells or CAR-T cells by not-fully known mechanisms. BCL-X_L_ targeting PROTACs seem to specifically inhibit TI-Tregs for immune activation. It is unknown whether a similar mechanism of action occurs in human cancers. Those results provide some rationale to combine these BCL-2 family inhibitors with immunotherapies.

Overall, inhibitors/degraders of the BCL-2 family kill cancer cells directly, which can further sensitize cancer cells to other therapeutics or can produce tumor antigens for activation of the tumor-immune cycle via DC-mediated antigen presentation. Pharmacological inhibition of the BCL-2 family is able to enhance CD8^+^ T cell activation via various mechanisms such as Treg depletion, which further enhances the killing of cancer cells that are sensitized by BCL-2 family inhibition.

## Transcription factors

### STAT3

STAT3 is a transcription factor and plays a role in transmitting signals from cell surface receptors to nuclear gene expression [67]. STAT3 is involved in and contributes to 10 of the 14 recently updated hallmarks of cancer [68, 69]. STAT3 is mainly activated by phosphorylation of tyrosine 705 (pTyr705) via IL-6 or other cytokine receptors, non-receptor or receptor tyrosine kinases such as those belonging to the JAK and Src family, as well as epidermal growth factor receptor and insulin growth factor 1 receptor [67, 70]. pTyr705 of STAT3 triggers its dimerization and translocation to the nucleus to activate specific genes including several oncogenes [71]. Other post-translational modifications of STAT3 include phosphorylation of serine 727 (pSer727) and the acetylation or methylation of several lysines [67, 72], most of which have been shown to affect STAT3 transcriptional activity. In normal cells, STAT3 activity is regulated to maintain a transiently active state, but its activity becomes dysregulated in cancer due to the atypical activation of many tyrosine kinases within cancer cells [71]. STAT3 is considered to be an oncogene and has long been a drug target of interest for cancer therapy [67, 68, 71–75].

**Figure 4.**
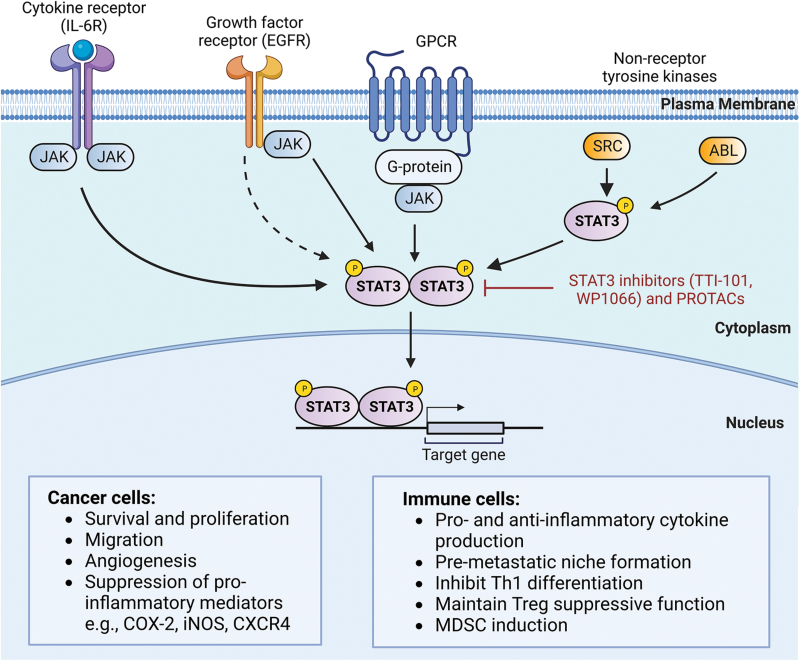
**STAT3 signaling and functions in cancer and immune cells.**Stimulation of cytokine receptors, growth factor receptors, G-protein coupled receptors (GPCRs), or non-receptor tyrosine kinases induces pTyr705 leading to dimerization of STAT3 via JAK-dependent or -independent pathways. STAT3 dimers translocate to the nucleus and transactivate genes regulating various functions in cancer and immune cells. STAT3 inhibitors, such as TTI-101 and WP1066, disrupt STAT3 dimerization leading to tumor suppression and immune activation. PROTACs against STAT3 have been shown to replicate those effects with greater efficiency and selectivity.

As SH2-domain-mediated dimerization is a critical step in STAT3 activation, most efforts have focused on developing STAT3 inhibitors that target the SH2 domain to interfere with the dimerization process (**Fig. 4**) [68, 71, 73–75]. Many STAT3-specific inhibitors have been developed with some of them including TTI-101, WP1066, pyrimethamine, OPB-33121, OPB-51602, and OPB-111077, e.g., entering clinical trials. OPB-33121 and OPB-51602 trials demonstrated the efficacy of STAT3 inhibition in cancer treatment [76, 77], but have to be terminated due to poor pharmacokinetics and toxicities [78]. In two genetic hepatocellular carcinoma (HCC) models, TTI-101 was able to halt HCC progression and remarkably, normalize liver function [79]. TTI-101 was recently granted fast-track designation for HCC based on the initial phase I data.

STAT3 also plays a role in immune modulation within the TME. Constitutive STAT3 activation suppresses proinflammatory mediators such as cyclooxygenase 2 (COX-2), inducible nitric oxide synthetase (iNOS), PD-L1, and C-X-C motif chemokine receptor 4 (CXCR4) [74]. Blocking STAT3 activity in tumor cells induces DC maturation and activation and the consequent T cell activation. TTI-101 seems to skew CD4^+^ T cells from IL-17-producing T-helper (Th)17 cells to IL-2-producing Th1 cells in non-tumor-bearing mice; the latter is well-known for its anti-cancer functions [80]. In the patient-derived xenografts, HCC using humanized mice or syngeneic HCC models, TTI-101 treatment enhances the anti-tumor effect of anti-PD-1 ICI therapy, further supporting the role of TTI-101 in modulating the TME [79]. In addition to mediating the cancer cell-immune crosstalk via cancer cell-intrinsic signaling pathways, STAT3-mediated immune suppression also occurs within different immune cell types [81]. The TTI-101-induced Th17 phenotype could be a direct influence of STAT3 inhibition in CD4^+^ T cell lineage commitment [79, 82, 83]. STAT3 also directly regulates forkhead box P3 (FOXP3) expression in Treg cells, hence maintaining the immune suppressive function of Treg cells [84]. WP1066, another STAT3 inhibitor in phase I clinical trial (ClinicalTrials.gov Identifier: NCT01904123), has been shown to directly enhance anti-tumor immunity by inhibiting TI-Treg function in mice bearing melanomas [85, 86]. IL-6 and GM-CSF—two upstream activators of STAT3—are shown to induce MDSCs; whereas this process can be inhibited by WP1066, leading to a reduction in MDSC number and PD-L1 expression (**Fig. 4**) [87].

While all the earlier generations of STAT3 inhibitors do not have exclusive selectivity toward STAT3, PROTAC-based STAT3 degraders exhibit good efficiency and selectivity with a neglectable effect on the degradation of other STAT family members [75, 88]. Two STAT3 PROTACs, SD-36 and SD-91, exhibit tumor-inhibitory effects *in vivo* and are safe in animals [75, 88]. SD-91, the CRBN E3 ligase-based STAT3 PROTAC, led to complete tumor regression in mouse xenografts of MOLM-16 acute myeloid leukemia (AML) [75, 88]. KT-333 is another highly selective STAT3-degrading PROTAC that is developed by Kymera Therapeutics. KT-333 is currently in a phase I study in adult patients with relapsed or refractory lymphomas, large granular lymphocytic leukemia, and solid tumors (ClinicalTrials.gov Identifier: NCT05225584). There is no report on STAT3-degraders in immune modulation, but further studies are warranted to thoroughly understand how these STAT3 degraders influence the TME and whether they can be combined with ICIs to improve therapeutic efficacy.

### NR4A1

**Figure 5.**
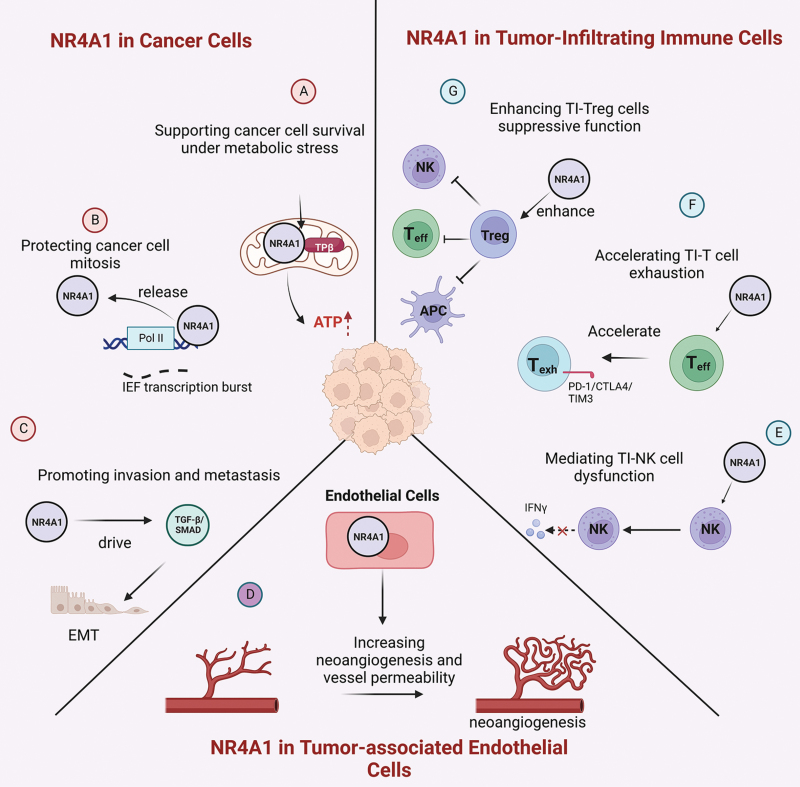
**NR4A1 protein in immune modulation and cancer therapy.**NR4A1 is an emerging target for cancer therapy due to its many roles in different cell types within the TME. (A-C) NR4A1 is known to promote cancer cell progression/metastasis via its pro-survival function under metabolic stress, cancer cell mitosis, and mediating gene expression involving invasion and metastasis. (D) NR4A1 has been shown to promote neoangiogenesis and vessel permeability. (E-G) NR4A1 has immune modulatory functions including the induction of natural killer (NK) cell dysfunction (E), promoting effector T cells (T_eff_) to T_exh_ cell state (F), as well as the immune suppression functions in TI-Tregs (G).

NR4A1 belongs to the steroid-thyroid hormone-retinoid receptor superfamily with two closely related members NR4A2 and NR4A3, all of which bind to nerve growth factor-induced clone B (NGFI-B) response elements (NBRE) [89]. The NR4A1 family has complex and opposing functions depending on the cancer type. In AML, it functions as a tumor suppressor [90, 91], and NR4A1-deletion facilitates AML development in mouse models [91]. In other cancers, NR4A1 promotes tumor progression via various mechanisms such as the promotion of epithelial-mesenchymal transition (EMT), invasiveness, and metabolic adaptation (**Fig. 5A–5C**) [92–102]. In cancer cells, translocation of NR4A1 from the nucleus to the mitochondrion has been shown to lead to apoptosis [103]. Mitochondrial NR4A1 is also known to bind to thyroid hormone receptor β and promotes cell survival of melanoma cells under metabolic stress [95]. Other studies have shown that NR4A1 is induced by oncogenic serine/threonine-protein kinase B-raf (BRAF) in melanoma cells and is involved in promoting the proliferation, survival, and invasiveness of melanoma cells [104]. BRAF inhibitor resistance can be mediated through the collective upregulation of several key transcriptional programs, some of which are regulated by NR4A1 [105].

NR4A1 has also been shown to play a role in endothelial cells within the TME (**Fig. 5D**). Knockout of NR4A1 in the host mice results in decreased angiogenesis in B16 mouse melanoma tumor models [106]. The impact of NR4A1 on neoangiogenesis has been confirmed in several other models as well [92, 107–109]. As neoangiogenesis often produces blood vessels with increased permeability, NR4A1 plays a critical role in regulating basal vascular permeability by increasing endothelial nitric-oxide synthase and downregulating several junction proteins involved in adherens junctions and tight junctions [102].

NR4A1 also plays a major role in immune cells in the TME (**Fig. 5E–5G**). NR4A1 and its family members have been shown to be critical in maintaining the exhausted state of CD8^+^ T cells; thus, targeting NR4A1 may reverse CD8^+^ T cell exhaustion which is a major focus of current immunotherapies [110, 111]. We have identified that NR4A1 is highly elevated in human TI-Tregs [112] and others have found NR4A1 expression in TI-Tregs from melanoma [113]. The role of NR4A1 in TI-Tregs is well-established in *NR4A1*^-/-^ mice where the NR4A1 family sustains the suppressive TI-Tregs [96, 114].

NR4A1 is an orphan receptor without any known physiological ligand. Several compounds have been found to bind NR4A1, either acting as antagonists or agonists such as a natural compound celastrol, cyclosporine B, and diindolylmethane analogs [115]. Some of these compounds have tumor-inhibitory effects, but none of them have entered clinical investigation yet [115]. 1,1-bis(3ʹ-indolyl)-1-(3-chloro-4-hydroxy-5-methoxyphenyl) methane (Cl-OCH3), one of the diindolylmethane analogs that can inhibit NR4A1, led to decreased expression of PD-L1 and reduction in TI-Treg cells using the 4T1 triple negative breast cancer model [116]. These compounds have adequate binding affinity to NR4A1 [115], hence providing good starting points for designing and synthesizing NR4A1 PROTAC degraders. We recently synthesized a panel of celastrol-based NR4A1 PROTAC degraders, some of which have outstanding efficiency in the degradation of NR4A1 in cancer cell lines as well as in syngeneic tumor models including B16 melanoma and MC38 colon cancer models. Further studies demonstrated that some of these compounds significantly inhibit tumor growth, mainly via immune modulations including the reduction of TI-Tregs and the induction of CD8^+^ T cells (Application number: PCT/US2021/048007). Further development of these compounds may have great potential for clinical translation to treat various solid cancer types.

### Conclusive remarks:

As an oncogene, STAT3 inhibitors/degraders can kill cancer cells directly or inhibit gene expression involving all other cancer hallmarks. Additionally, blocking STAT3 increases the proinflammatory mediators within cancer cells, which in turn can potentially induce DC activation [74, 80]. STAT3 inhibitors have been shown to inhibit PD-L1 and reduce the number of TI-Tregs [84–86], which can collectively reverse T cell inhibitory pathways. Early-phase clinical trials have been unfavorable for STAT3 inhibitors, likely due to the selectivity to other STAT family proteins with contrasting effects in tumorigenesis and severe toxicity including peripheral neuropathy and elevated serum lactate in the case of OPB-51602 [77]. Future efforts should focus on the development of more specific inhibitors to nuclear STAT3 activation with improved pharmacodynamics and pharmacokinetics, while sparing the intact mitochondrial functions of STAT3 [77]. STAT1 shares very significant structural similarity with STAT3, but it has known effects on immune activation and tumor suppression. Some recently developed inhibitors or PROTACs, such as TTI-101, KT-333, SD-36, or SD-91, exhibit selectivity to STAT3 and are expected to have a better therapeutic effect. Further studies in combination with ICIs or chemotherapeutics are warranted for further establishing the therapeutic efficacy toward different cancers.

Current targeting of NR4A1 is still at pre-clinical stages, but the genetic evidence has strongly established NR4A1 as a good target for immune therapeutics to eliminate Tregs and/or rejuvenate T_exh_ cells, as well as targeting the invasive and metabolic pathways in cancer cells. Clinically targeting NR4A1 is expected to have an impact on immunogenic tumors either alone or in combination with other ICIs.

## Multifunctional epigenetic modulators

### HDAC family members

**Figure 6.**
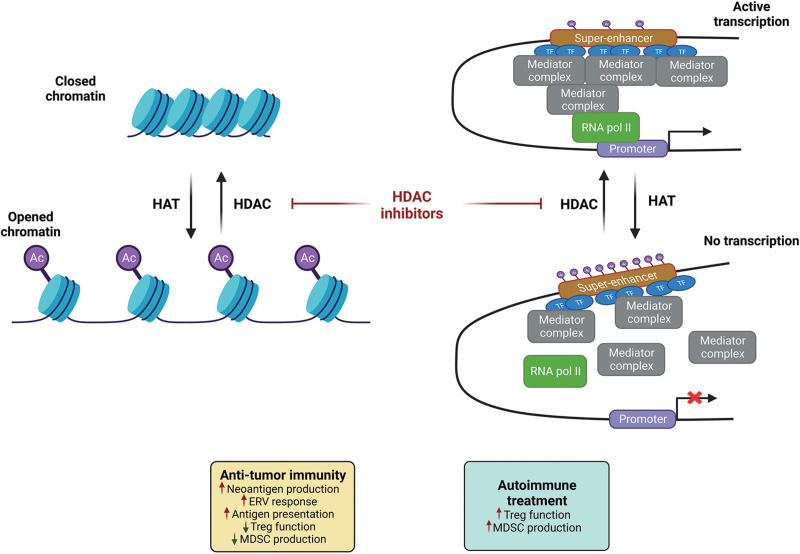
**HDAC family in immune modulation and cancer therapy.**Eleven human HDAC family members are categorized into four groups. Most HDACs can deacetylate histones that control chromatin structures and the cooperation between super-enhancer and promoter, leading to consequent changes in gene expression. Some of them have direct protein substrates other than histones. HDAC inhibitors are mostly non-selective and can inhibit HDACs within the same group and even across groups. Several inhibitors have been approved for the clinical treatment of hematopoietic cancers, but none exhibit satisfactory therapeutic efficacy in solid cancers. Different HDAC proteins may have opposing immune-modulatory functions, and specific inhibitors are needed and required for activating anti-cancer immunity.

In humans, 11 zinc-dependent HDAC proteins can remove acetyl groups from lysines, one of the main post-translational protein modifications particularly important for histones [117]. Human HDACs can be categorized into four classes based on sequence similarity and sub-cellular localizations. HDAC1, HDAC2, HDAC3, and HDAC8 belong to class I; HDAC4, HDAC5, HDAC7, and HDAC9 belong to class IIa; HDAC6 and HDAC10 are within class IIb; and HDAC11 is the sole member of class IV. Class III are sirtuins 1–7 using nicotinamide adenine dinucleotide (NAD)^+^ as a cofactor instead of zinc [117]. In addition to deacetylating histones, other protein targets are also reported for HDAC-mediated deacetylation, such as tumor protein 53 (p53), E2F, ataxia telangiectasia mutated, chromatin assembly factor 1, DNA methyltransferase 1, and LSD1 for HDAC1/2; STAT3 for HDAC3; structural maintenance of chromosomes protein 3 (SMC3), p53, estrogen-related receptor α, AT-rich interactive domain-containing protein 1A, etc. for HDAC8 [117]; FOXP3 for HDAC7/9 [118]. The histone deacetylation is normally coupled with other epigenetic processes such as histone or DNA methylation, leading to the regulation of chromatin condensation, the cooperation between super enhance and promoter, and the regulation of gene expression (**Fig. 6**) [119]. HDACs are commonly overexpressed in many cancers and have long been thought of as therapeutic targets. It has been more than 30 years since the discovery of the first HDAC inhibitor. Four of the HDAC inhibitors have been approved by the FDA, including vorinostat, belinostat, romidepsin, and panobinostat, along with the approval of a benzamide analog (tucidinostat) for cancer therapy. All these approvals are for hematologic tumors, such as cutaneous T-cell lymphoma and multiple myeloma, and have been extensively summarized in a recent review [117]. HDAC inhibitors are also known for their off-target toxicities. In a phase I trial for romidepsin, there were five cases of cardiac arrest in patients [120]. Other studies have indicated other cardiac toxicity including sinus tachycardia and atrial fibrillation [121]. All HDAC inhibitors as single-agent therapeutics are ineffective in treating solid cancers [122]. This raises the question whether a combination with other anti-tumor agents, in particular immune modulatory agents, would work for treating patients with solid cancers. The potential combinations of HDAC inhibitors with other antitumor agents were also recently discussed [123].

Several HDACs are known to modulate immune functions (**Fig. 6**). In relation to cancer cell-intrinsic immune modulations, earlier studies demonstrated that HDAC inhibitors impact the tumor immune microenvironment and enhance the antitumor efficacy of ICIs in preclinical studies [124–126]. The class I HDAC-specific inhibitor entinostat induces immune editing of tumor neoantigens, resulting in a strong anti-tumor immune response [127]. The cancer-testis antigen (CTA) family of proteins can be re-activated in tumors via epigenetic modifications, which are also the targets of panobinostat-mediated HDAC inhibition [128]. ERVs are another source of tumor antigens that can be re-activated via epigenetic regulation. Vorinostat and romidepsin can both enhance these tumor antigens [129]. Several HDAC inhibitors have been shown to enhance the antigen-presenting capacity of DCs via various mechanisms, which ultimately led to anti-tumor immune responses. This has recently been reviewed [130].

FOXP3 is a Treg lineage marker and a direct substrate of HDAC7/9 [118, 131]. HDAC9 is particularly important in FOXP3-dependent IL-2 suppression and Treg lineage commitment [131]. A pan-HDAC inhibitor (sodium butyrate) was able to enhance Treg cell function by promoting FOXP3 binding to chromatin [132]. HDAC6, a class IIb HDAC, plays a similar role in the Treg function. HDAC6 knockout or HDAC6 specific inhibitors (tubacin and tubastatin A) increase FOXP3 expression and enhance Treg suppressive functions [133]. These data have generated interest in using HDAC inhibitors to treat autoimmune diseases and transplant rejections [134]. HDAC3, however, seems to have an opposite function and associates with FOXP3 to maintain the function of both thymic/natural and induced Tregs. HDAC3-deficient mice died from autoimmunity by 4–6 months of age, which can be rescued by the adoptive transfer of wild-type Tregs [135]. A separate study confirmed that the CoREST complex—including CoREST1/2/3 (encoded by RCOR1/2/3), LSD1, and HDACs—is critical in maintaining the function of Treg cells and inhibition of CoREST impairs the suppressive function of Tregs [136]. The complex roles of different HDACs on Tregs require careful consideration when choosing the right HDAC-specific inhibitors. Most reported preclinical or clinical data showed that HDAC inhibitors could reduce immune-suppressive Tregs [137–141]. This decline in Treg cells was associated with response to therapy [138, 142]. As such, it is not surprising to see that HDAC inhibitors were able to enhance the ICI therapeutic efficacy [139, 143].

The response of MDSCs to HDAC inhibitors follows a similar pattern to that of Treg cells. Initial observation supports that HDAC inhibition by trichostatin A facilitated the expansion of MDSCs [144]. Tumor studies, however, have shown supportive data that HDAC inhibitors inhibited or depleted MDSCs in primary tumors [139, 140, 145–149] and premetastatic niches [150]. HDAC inhibitors were also shown to have different effects on gMDSCs versus mMDSCs [145, 151], and some were able to reprogram TAMs from an M2-like phenotype to an M1-like phenotype [151].

The development of conventional isozyme-specific HDAC inhibitors remains challenging as the catalytic domain of HDACs is highly conserved. HDAC inhibitors of different chemical structures in addition to hydroximic acids, benzamides, and cyclic peptides have been discovered; and some of these, such as the hydrazide class of inhibitors, show improved selectivity [152–155]. Further improvement of isozyme selectivity can be achieved by converting the hydrazide inhibitors to PROTACs [156]. HDAC inhibitors of hydroximic acid, benzamide, and other classes have also been converted to PROTACs [157–162]. The potential of HDAC degradation by PROTACs for enhancing cancer immunotherapy has recently been reviewed [163]. While earlier clinical trials of HDAC inhibitors show potential clinical benefit for patients with breast cancer [164], the role of HDAC inhibitors/degraders in immune modulation may provide a unique opportunity for some of those specific inhibitors/degraders—which have sufficient therapeutic windows and limited toxicity—to sensitize some solid cancers for ICI-based therapy. There have been many active phases 1 and 2 clinical trials for the combination of HDAC inhibitors and ICIs. It is expected that some of these trials may advance into phase 3 and get approval for cancer treatment.

### BET proteins

**Figure 7.**
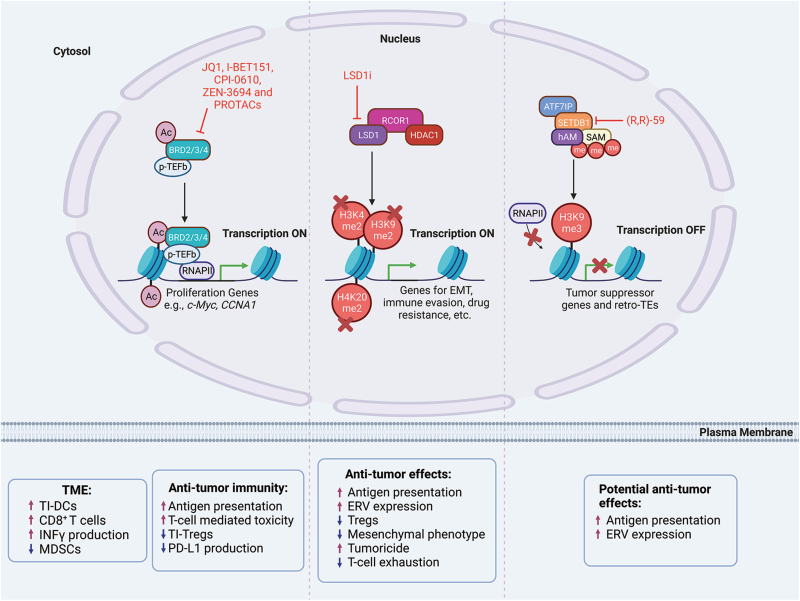
**Several epigenetic modulators in immune modulation and cancer therapy.**Epigenetic modulators have been an active field of research for cancer therapy. Left: BET family proteins, with BRD4 as an example, are known to facilitate the cooperation between acetylated histones within the super-enhancers (SE) and promoters of several oncogenes, such as *c-MYC* and *CCNA1*. Inhibitors/degraders shortcut the cooperation between SE and promoters, which results in the repression of oncogene expression. Middle: CoREST complex consists of LSD1, RCOR1/2/3, and HDAC1/2 and coordinates between histone deacetylation and methylation. Downstream targets of LSD1 include genes involved in EMT, immune evasion, or drug resistance. LSD1 inhibitors lead to epigenetic silencing of these genes by enhancing histone methylation, with ultimate tumor inhibitory function via either cancer cell-intrinsic mechanisms or via immune activation. Right: SETDB1 is a relatively novel target for cancer immunotherapy but could pose as an effective target since its activity leads to histone methylation at histone 3 lysine 9 (H3K9). In cancer, this leads to gene silencing with histone mark H3K9me3 at tumor suppressor genes and/or ERV (Retro-TEs). Inhibitors of SETDB1 can potentially reverse the histone marks which would result in the expression of tumor suppressive factors or ERVs. ERV proteins can result in the activation of interferon-related pathways and serve as tumor antigens for immune activation.

The mammalian BET family of proteins consists of four members, bromodomain-containing protein (BRD) 2 (BRD2), BRD3, BRD4, and BRDT that regulate transcription through epigenetic interactions between bromodomains and acetylated histones [165]. These interactions result in an upregulation of cellular proliferation genes such as *MYC* and *CCNA1* (Cyclin A1) (**Fig. 7**, left) [166]. Specifically, BRD4 has become a well-established target for cancer therapy [167]. Multiple studies have demonstrated that BET inhibitors (BETi) are capable of attenuating tumor proliferation [166, 168]. However, the efficacy of pan-BET selective inhibitors, such as JQ1 and I-BET151, is still obstructed by acquired drug resistance in clinical trials [166]. It was also shown that long-term dosing with BETi insufficiently suppresses c-MYC expression and leads to an accumulation of BRD4 [169]. CPI-0610 is a pan-BET inhibitor and shows promising results in treating patients with myelofibrosis and related neoplasms [170, 171]. ZEN-3694 is a potent second-generation pan-BET inhibitor with oral bioavailability, showing some promising results in several earlier-phase clinical trials for hormone-responsive cancer types [172–174]. There are currently eight clinical trials using ZEN-3694 actively recruiting cancer patients with various cancer types. Common side effects of these BET inhibitors include dose-limiting thrombocytopenia, anemia, neutropenia, gastrointestinal events, and fatigue [172–174].

As BET proteins are known for their functions in immune regulation [175], it is not surprising to see that BET inhibitors can modulate the TME. JQ1—a first-generation pan-BET inhibitor—is reported to increase the frequency of TI-DCs, and CD8^+^ T cells, as well as to induce the production of interferon γ. Simultaneously, it has been shown to reduce immune suppressive MDSCs in mesothelioma-bearing mice [176]. A separate study supports the role of JQ1 in activating anti-tumor immunity via several different mechanisms, including (i) increasing H2Kb-restricted tumor antigen presentation, (ii) enhancing T cell-mediated toxicity to cancer cells, and (iii) reducing the frequency of TI-Tregs [177]. Another level of T cell activation is through the rejuvenation of T_exh_ which express PD-1 and/or other immune checkpoints. PD-L1 is the ligand for PD-1 and can induce T-cell exhaustion. PD-L1 is a direct target of BET proteins and JQ1 inhibits PD-L1 production [177–180]. The immune modulatory function of JQ1 enhances the efficacy of ICI therapy in a mouse model of colorectal cancer [177] and K-Ras mutant non-small cell lung cancers [181].

PROTAC-based BET protein degraders are also under development for clinical translation. Several groups have developed BET protein degraders using JQ1 as a warhead, including dBET1 [182], MZ1 [183], dBET21 (patent number: WO2017007612A1), ARV-771 [184] and several other compounds from a recent study [185]. ARV-825 uses a different warhead OTX015 [185]. Most of these are still in the early stage of research development, and none has entered clinical trials. These have been tested in multiple cancer models including castration-resistant prostate cancer [186], triple-negative breast cancer[187], and leukemia [188]. Most of the tested PROTACs are not specific to BRD4 and can also degrade BRD2 and BRD3. As such, evidence shows that increased drug toxicity and off-target side effects may result from the simultaneous degradation of the other BRD proteins [189, 190]. The immune-modulatory functions of these PROTACs are yet to be tested in preclinical models.

### LSD1

Another previously mentioned epigenetic modulator is LSD1 which demethylates histones and activates gene expression [191–193]. LSD1 forms a complex with HDAC1/2 along with the scaffolding proteins RCoR1/2/3 known as the CoREST complex. As a flavin-dependent amine oxidase, LSD1 demethylates mono-methylated or di-methylated Lys4 and Lys9 at histone H3, and Lys20 at H4 (H3K4me1/2, H3K9me1/2, and H4K20me1/2) [191–194]. Demethylation of chromatin substrates by LSD1 requires RCoR1 [195], and they co-occupy 94.1% of all genome-wide binding sites of LSD1 [196], suggesting that the epigenomic function of LSD1 is largely associated with the CoREST complex. Notably, the demethylase and deacetylase activities of CoREST are interdependent and work in concert for efficient histone deacetylation and demethylation [191, 197–199]. Because chromatin with acetylated lysines and methylated H3K4 is typically associated with gene activation, enzymatic activities of CoREST cooperate synergistically for gene silencing [200–202]. Notably, histone deacetylation and LSD1-mediated demethylation also activate gene expression [119, 192, 193, 203–205]. Functionally, LSD1 promotes EMT, stemness, metastasis, immune evasion, and drug resistance [206–211], providing a premise for LSD1 as a potential drug target in oncology. Several LSD1 inhibitors (LSD1i) have been developed (**Fig. 7**, middle), some of which are in clinical trials but have yet to be approved by the FDA. Studies have shown that concomitant inhibition of LSD1 and HDAC appears to be more effective at killing cancer cells than HDAC inhibition alone [200, 212]. LSD1 also confers resistance to cancer immunotherapy [209, 210, 213]. LSD1 inhibition increases gene expression of the MHC-I antigen presentation pathway [214] and ERVs [209]. LSDi attenuates the immune-suppressive effects of Tregs and enhances antitumor immunity [136]. Combinatory chemotherapy and LSD1i therapy are highly effective in tumor suppression, apparently through downregulating genes underlying mesenchymal phenotypes while promoting an innate, M1 macrophage-like tumoricidal immune response [215, 216]. Furthermore, LSD1 is implicated in T-cell exhaustion, and LSD1 inhibition reinvigorates T cells and improves cancer immunotherapy [207, 211].

### SETDB1

Cancer cells overexpress SETDB1 to regulate the methylation of histones to facilitate cancer evasion from immune surveillance. SETDB1 methylates lysine residues present on histone 3 lysine 9 (H3K9) to inhibit gene transcription of specific regions containing transposable elements and ERV genes [217–220]. This mechanism prevents the expression of broad domains of targetable antigens that can be recognized by effector T cells within the TME thereby mediating immune escape [217, 218, 220]. SETDB1 has been implicated in reducing the effectiveness of ICIs [217, 218] as well as radiotherapy [221]. The importance of SETDB1 in immune modulation has just begun to surface, and pharmacological inhibitors of SETDB1 are at a very earlier stage of development, including a competitive inhibitor (R, R)-59 with a *K*_*d*_ of 88 nM [222]. The therapeutic effect of these inhibitors is yet to be determined (**Fig. 7**, right).

### Conclusive remarks:

The HDAC family has been one of the centered protein families for drug development with some success; however, toxicity will be the major hurdle for HDAC inhibitors to be used in solid cancers, which normally require higher and longer dosing regimens to reach clinical efficacy. HDAC members share high sequence and structural similarities. All FDA-approved inhibitors can inhibit several HDACs. Current efforts should be focused on developing more specific inhibitors to disease-relevant HDAC members. HDACs are involved in several different stages of the tumor-immune cycle, including antigen induction/presentation, immune suppressive function via Tregs or MDSCs, or induction of immune checkpoints. It is expected that some of the current clinical trials combining HDAC inhibitors and ICIs will produce some positive data in cancer treatment. Like the HDAC family, the other aforementioned proteins, such as the BET family, LSD1, SETDB1, or other proteins within the same complexes, are expected to have multiple immune-modulatory functions within the TME. Current clinical focus on these targets—in the near future—may provide alternative therapeutics for treating broader cancer types than current ICI-responsive cancers, or for treating ICI-resistant cancers. Targeting these epigenetic factors seems to have on common effect on the re-expression of ERVs within the tumor cells that can serve as tumor antigens for DC activation and T cell priming, which is very attractive in switching a cold immune TME to a hot one and further sensitives cancers to ICI-based cancer immunotherapy.

## Conclusion and perspective

### Releasing endogenous tumor antigens for initiating the tumor-immune cycle

Among the many steps of T cell activation during the tumor-immune cycle, ICIs work efficiently to revert an exhausted T cell state in immunogenic cancer types such as melanoma and clear cell renal cell carcinomas. Targeting other steps in the tumor-immune cycle and/or increasing immunogenicity can increase the efficacy of ICIs. Many factors contribute to immunogenicity within different cancer types, with the mutational load being one of the most important factors [223]. This is the rationale behind why pembrolizumab is approved by the FDA to treat microsatellite instability-high (MSI-H) or mismatch repair deficient (dMMR) solid tumors, irrelevant of cancer type [224]. Elevated mutational burdens—including those tumors bearing MSI-H or dMMR—are associated with an increase in neoantigens, which can lead to T cell-intrinsic activation programs that allow for the recognition of neoantigens [223]. Other tumor antigens, such as self-antigens or ERV genes, are normally epigenetically silenced under physiological conditions—a similar mechanism related to several tumor suppressor genes [225]. Pathological changes, such as cancer, can have mutations in those epigenetic regulators, with DNA methyltransferase 3A being an outstanding example whose mutation leads to AML [226]. Therapeutic inactivation of some epigenetic proteins may lead to the re-expression of tumor suppressor genes, aiding in tumor inhibition [227], or the expression of some immunogenic self-antigens and/or ERV antigens [129, 209, 217–220]. Though neoantigens are more intrinsic to the nature of individual cancer, self-antigens or ERV antigens can be therapeutically induced by targeting epigenetic modulators, including the aforementioned HDACs, SETDB1, LSD1, and BET family [129, 209, 217–220]. Access of DCs or other APCs to these antigens, however, can be a tricky process. Vaccination is a strategy to promote such processes aiming at priming and activating T cells in a tumor antigen-specific manner [228]. Therapeutic approaches, such as chemotherapy and/or radiotherapy, can induce immunogenic cell death, providing multiple complex processes that can lead to simultaneous DC activation and tumor antigen access [229]. This could be the fundamental reason why chemotherapy significantly increases the response rate of cancer patients to ICIs in advanced clinical trials [230]. Combinatory radiotherapy with ICIs may also increase patient response rate [231]. The caveat for chemotherapy or radiotherapy is that activated T cells, or other lymphocytes, are sensitive to these treatments and sometimes more sensitive than cancer cells [232, 233]. Other more targeted approaches, such as the BCL-2/BCL-X_L_ inhibitors/degraders, can kill cancer cells that depend on those proteins for survival [44, 63, 64]. Although these targeted therapies have been known to induce apoptosis rather than immunogenic cell death, there are some reports supporting that apoptotic cancer cells are capable of presenting antigens for DC presentation [234–236]. The combination of different therapeutics with ICIs has to be considered carefully to balance T cell priming/activation versus T cell elimination for further clinical research.

### Combinatory targeting immune checkpoints involving the initial priming and activation stages

In the classic tumor-immune cycle, the next step involves T-cell activation within the lymph nodes. This is where T cells are primed and activated by DCs; the anti-CTLA-4 antibody (ipilimumab) could be partially effective at this stage by blocking CTLA-4 and CD80/CD86 binding and releasing co-stimulatory signals to T cells (**Fig. 1**, signal 2) or by promoting endocytosis of these co-stimulatory signals [237, 238]. Another interesting immune checkpoint, V-domain Ig suppressor of T cell activation (VISTA), at the priming stage was recently reported [239]. VISTA may be potentially targeted for cancer immunotherapy. Combinational targeting of both the priming stage immune checkpoints and the T_exh_ checkpoints (such as PD-1 or PD-L1) may have better T cell activation potential at various stages. The combination of ipilimumab and pembrolizumab or nivolumab (anti-PD-1) has already gotten several FDA approvals for different cancer types.

### Targeting immune suppressive cell types including Tregs and MDSCs

Compared to ICIs in cancer treatment, research focused on targeting immune suppressive cells, such as TAMs, MDSCs, and Tregs in the TME is significantly lagging in the clinical development. This is a bit surprising considering these cell types are well-known for inhibiting T cell function, and some are the primary contributors to resistant mechanisms against ICIs. Efforts to inhibit these cell types have also focused on cell surface functional proteins, such as the anti-CD25 antibody and variants for blocking Treg function or CCR2 inhibitors for TAM or MDSC recruitment. Adaptation of these cells to the TME, however, may require the function of some common and important intracellular proteins, such as survival factors (e.g., BCL-2 or BCL-X_L_), transcriptional factors (e.g., the NR4A1 family and STAT3), as well as some epigenetic alterations (e.g., SETDB1, LSD1, BET proteins, and the HDAC family). This review emphasizes these factors since they also have important for cancer cells, but do not negatively influence effector cell functions. For example, we found significant elevation of BCL-X_L_ in TI-Tregs, and PROTAC-mediated degradation of BCL-X_L_ led to decreased tumor growth along with increased T cell activation [45]. The BCL-X_L_-targeting PROTACs have a better therapeutic window due to the selective degradation of BCL-X_L_ within cancer cells and TI-Tregs, but not in platelets—the common dose-limiting toxicity for BCL-X_L_ inhibitors. CD8^+^ T cells, however, mainly rely on BCL-2 for survival and are not eliminated during PROTAC treatment. The therapeutic effect of BCL-X_L_ PROTACs may be more efficient in cancer types where overexpression of BCL-X_L_ predicts a worse prognosis, such as clear cell renal cell carcinoma, glioma, cervical squamous cell carcinoma, etc. [44, 63, 64]. The pan-cancer targeting potential for BCL-2 or BCL-X_L_ inhibitors/degraders gives these drugs a broader patient population and combinational therapy capacity with chemotherapies, radiotherapies, or immunotherapies.

### Targeting multi-functional proteins involving different components within the TME

NR4A1, STAT3, and BET proteins are three examples of multifunctional proteins that promote cancer progression via both cancer cell-intrinsic transcriptional programs and lineage-specific immune modulations. NR4A1 is a known resident T cell marker [240, 241] and is significantly elevated in TI-Tregs as well as in exhausted T cells. Outstanding genetic data show its critical role in maintaining those cells in their immune suppressive and inactive state, respectively [110–112]. Due to different genomic architectures within the various cell lineages, the same transcription factor can lead to different gene expression programs. It is challenging, but attainable, to identify those transcription factors that have lineage-specific and tumor-promoting functions. For example, NR4A1 also plays a role in CD16^+^ patrolling monocytes that are known to inhibit metastasis in a B16 melanoma model [242]. For NR4A1 inhibitors/degraders to work efficiently, it is important to know the dominant immune cells present within the TME and what functions they have in tumor promotion. Another potential concern is the on-target or off-target toxicities due to protein multifunctionality. BET inhibitors/degraders are not selective to individual BET proteins, and the observed toxicity may be due to the simultaneous inhibition of multiple BET proteins. Selective inhibitors/degraders may be achievable to avoid this toxicity caused by broader targets. STAT3 is an important oncogenic target, but most STAT3 inhibitors can block STAT1, a known interferon downstream effector protein involved in activating anti-tumor immunity. The therapeutic effect of STAT3 inhibitors may be negated due to the dual inhibition of STAT3 and STAT1. The newly developed STAT3-PROTAC seems to avoid STAT1 degradation and could be a potential drug candidate for targeting STAT3. Due to the embryonic lethality phenotype of germline STAT3 deletion in mice [243], STAT3 inhibitors/degraders should be used with caution in children and during pregnancy due to potential toxicity during early development.

In addition to those discussed above (Table 1), there are many other potential transcription factors or epigenetic regulators that can potentially be targeted for cancer immunotherapy. Most drug targets have been primarily studied in the context of cancer-cell intrinsic functions. The development of these targets’ inhibitors/degraders in cancer therapy should be accompanied by in-depth research related to their capacity for on-target or off-target immune modulations, as well as considering cancer type-specific and TME-specific responses for different therapeutic targeting of these molecules.

**Table 1.** Inhibitors and degraders of multifunctional protein targets as emerging therapeutic targets for cancer immunotherapy

Inhibitor or degrader	Target protein	Cancer type/model	Side effects	Reference
ABT-737	BCL-2, BCL-W, BCL-X_L_	Small and non-small cell lung cancer	Unknown	[43, 47]
ABT-263	BCL-2, BCL-W, BCL-X_L_	Lymphoid malignancies, solid tumors	BCL-X_L_ inhibition: thrombocytopenia BCL-2 inhibition: T-cell lymphopenia	[48–52]
ABT-199	BCL-2	Hematologic cancers	Diarrhea, neutropenia, fatigue, upper respiratory tract infection, cough, and tumor lysis syndrome	[53–58]
WEHI-539	BCL-X_L_	BCL-X_L_-dependent cancers	Thrombocytopenia	[60]
A-155463	BCL-X_L_	BCL-X_L_-dependent cancers and small cell lung cancer	Unknown	[60]
A-1331852	BCL-X_L_	BCL-X_L_-dependent cancers	Unknown	[61]
*DT2216	BCL-X_L_	BCL-X_L_-dependent cancers	Reduced toxicity to platelets	[44, 63, 64] NCT04886622 (clinical trial #)
*PZ15227	BCL-X_L_	BCL-X_L_-dependent cancers	Reduced toxicity to platelets	[45, 63]
TTI-101	STAT3	Hepatocellular carcinoma (HCC)	Unknown	[79]
WP1066	STAT3	Brain cancers and metastatic melanoma	Unknown	[85, 86] NCT01904123 (clinical trial #)
*SD-36	STAT3	Acute myeloid leukemia (AML)	Not reported	[75, 88]
*SD-91	STAT3	Acute myeloid leukemia (AML)	Not reported	[75, 88]
*KT-333	STAT3	Relapse or refractory lymphomas, large granular lymphocytic leukemia, and solid tumors	Not reported	NCT05225584 (clinical trial #)
NR4A1 PROTAC	NR4A1	Solid tumors	Not reported	PCT/US2021/048007 (patent)
CPI-0610	pan-BET	Myelofibrosis and related neoplasms	Thrombocytopenia, anemia, neutropenia, gastrointestinal events, and fatigue	[170, 171]
ZEN-3694	pan-BET	Hormone-responsive cancers	Thrombocytopenia, anemia, neutropenia, gastrointestinal events, and fatigue	[172–174]
JQ1	pan-BET	Colorectal and K-Ras mutant non-small cell lung cancers	Thrombocytopenia, anemia, neutropenia, gastrointestinal events, and fatigue	[175–181]
I-BET151	pan-BET	Hematological malignancies, breast cancer, glioma, melanoma, neuroblastoma, and ovarian cancer	Thrombocytopenia, anemia, neutropenia, gastrointestinal events, and fatigue	[166]
*dBET1	BET	MV4-11 (AML) and DHL4 (lymphoma)	Not reported	[182]
*MZ1	BET	HeLa (cervical cancer) and U2O2 (osteosarcoma)	Not reported	[183]
*dBET21	BET	MV4-11, SUM149 triple-negative breast cancer (TNBC), DHL4, and SEMK2 (B-cell leukemia)	Not reported	WO2017007612A1 (patent)
*ARV-771	BET	Castration-resistant prostate cancer, TNBC, and leukemia	Not reported	[184, 185]
*ARV-825	BET	Castration-resistant prostate cancer, TNBC, and leukemia	Not reported	[184, 185]
(R,R)-59	SETDB1	HEK293T (human embryonic kidney) and THP-1 (human acute monocytic leukemia)	Not reported	[222]
LSD1i	LSD1	Small lung cancer cells (SCLC) and AML	Not reported	[136, 200, 207, 209, 211, 212, 214–216]
**Vorinostat**	HDAC1/2/3/6	Cutaneous T-cell lymphoma and breast cancer + pembrolizumab (tamoxifen)	Diarrhea, nausea, loss of appetite, weight loss, etc.	[117, 129]
**Belinostat**	Class I, Class IIa, and Class IIb HDACs	Peripheral T-cell lymphoma	Diarrhea, nausea, loss of appetite, weight loss, etc.	[117]
**Romidepsin**	HDAC1/2	Cutaneous T-cell lymphoma and peripheral T-cell lymphoma	Diarrhea, nausea, loss of appetite, weight loss, etc.	[117, 120, 121]
**Panobinostat**	HDAC1-11	Multiple myeloma and myelodysplastic syndrome	Diarrhea, nausea, loss of appetite, weight loss, etc.	[117]
**Tucidinostat**	HDAC1/2/3/10	Peripheral T-cell lymphoma, B-cell lymphoma, breast cancer, and soft-tissue sarcoma	Mild rash and some fatigue	[117]
Entinostat	HDAC1 and HDAC3	Breast cancer and renal carcinoma	Hypophosphatemia, anemia and fatigue in patients with solid tumors	[127]
Trichostatin A	HDAC1, HDAC3, HDAC4, HDAC6, and HDAC10/3/4/6/10	Ovarian, TNBC, colorectal, and cervical cancer	Not reported	[144]

Key: NA, Not Applicable; *Indicates a protein degrader; Bold, FDA approved.
